# Role of dyslipidemia in preeclamptic overweight pregnant women

**Published:** 2012-03

**Authors:** Seyede Hajar Sharami, Azita Tangestani, Roya Faraji, Ziba Zahiri, Azam Amiri

**Affiliations:** 1 *Reproductive Health Research Center, Department of Obstetrics and Gynecology, Guilan University of Medical Sciences, Guilan, Iran.*; 2 *Reproductive Health Research Center****, ****Guilan University of Medical Sciences, Guilan, Iran.*

**Keywords:** *Overweight*, *Pre-eclampsia*, *Dyslipidemia*

## Abstract

**Background: **Obesity is an independent risk factor of preeclampsia with unknown mechanism and hyperlipidemia might be a probable case of it.

**Objective:** The objective of this study was to determine the role of hyper-triglyceridemi in association with high prepregnancy body mass index and the risk of preeclampsia.

**Materials and Methods:** The authors conducted this case-control study of 42 preeclamptic and 41 normotensive overweight pregnant women. The two groups were comparable with respect to age, gestational age, and body mass index. Blood samples were collected at the time of diagnosis of preeclampsia, after 14 hour fasting to determine plasma lipid concentrations. Enzymatic photometric tests were used to determine lipid profile. Data was analyzed with independent “t-test”, Chi-square and one-way ANOVA and post HOC Tukey HSD test. The statistical significance was set at 0.05 levels.

**Results:** In the subjects with preeclampsia, serum triglyceride and total cholesterol levels were significantly increased and plasma HDL-cholesterol concentrations were decreased compared with the controls, (p<0.05), but plasma LDL cholesterol levels didn’t differ between the two groups. Women who developed severe preeclampsia had higher concentrations of TG and cholesterol and lower levels of HDL compared to noromotensive group. Mean TG: 375.16 vs. 202.85, p<0.001, Mean cholesterol: 245.64 vs. 214.32, p=0.04, Mean HDL: 40.80 vs. 48.95, p=0.03).

**Conclusion:** We noted that dyslipidemia, particularly hypertriglyceridemia was highly correlated with prepregnancy high BMI in preeclamptic women. These findings continue to support a role for dyslipidemia in BMI related preeclampsia.

## Introduction

Preeclampsia is a pregnancy-specific disease that present with new-onset hypertension and proteinuria after 20 weeks gestation. It involves 5-8% of pregnancies and it is leading cause of maternal and neonatal mortality and morbidity (1). Diagnostic criteria’s of preeclampsia are BP≥140/90 after 20 weeks of gestation and proteinuria (>300 mg/24h or ≥1+ in urine dipstick). 

In addition, women with a history of preeclampsia seem to be at an elevated risk for cardiovascular disease later in life (2). Risk factors for development of preeclamsia include nulliparity, extremes of maternal age (>35 years or <15 years), obesity, diabetes mellitus, history of renal disease or chronic hypertension, multiple gestation, and African American race (3). However various factors are implicated in the pathogenesis of preeclampsia including genetic, immune, vascular and oxidative stress, yet its etiology remains unclear, and little is still known about the pathogenesis of preeclampsia, making its prevention an ongoing challenge (4). 

Endothelial dysfunction is the most acceptable theory for etiology of preeclampsia. In this disorder due to deposition of fibrin and platelet and accumulation of lipid-laden microphages within vascular bed, placental perfusion is reduced (5). These findings have lead to the hypothesis that disorders of lipid metabolism might be a major cause of endothelial dysfunction. 

Pregnancy is associated with physiologic hyperlipidimia, and in normal pregnancy, this feature is not atherogenic that is believed to be under hormonal control (6). Women who develop preeclampsia, experience more dramatic lipid changes compared with normotensive women. 

Also patients with hyperlipidemia, especially hypertriglyceridemia have a higher incidence of and are prone to develop more severe cases of preeclampsia (7). Most, although not all studies have shown a dyslipidemic pattern of increased triglycerides, cholesterol, low density lipoprotein (LDL) and decreased high density lipoprotein (HDL) concentrations in preeclampsia (8). Obesity or high maternal prepregnancy body mass index (BMI) is a validated, independent risk factor for the development of placental endothelial dysfunction and preeclapsia, but the mechanism of how it imparts increased risk is not completely understood (9). 

High maternal BMI or obesity is one of the risk factors of preeclampsia and the risk of preeclampsia rose strikingly from a pregnancy BMI of 15 to a BMI of 35, such that, compared with a BMI of 21, the risk of preeclampsia was approximately doubled at a BMI of 26, and tripled at a BMI of 30. Hyperlipidemia and inflammation are two mechanisms that hypothesized for BMI related preeclampsia but not only this factors but also others, are not fully tested (10). 

There is lipid concentration alternation in overweight patient and this phenomenon is one of the characteristic features of preeclampsia (11, 12). Therefore hyperlipidemia might be part of a cause pathway through which obesity predisposes to preeclampsia. Because an elevated BMI and hyperlipidemia are major risk factors of preeclampsia, a more precise estimate of the association between maternal prepregnancy BMI, dyslipidemia and preeclampsia is needed. Accurately quantifying the relation of prepregnancy maternal obesity, hyperlipidemia and the risk of preeclampsia may better identify those at highest risk. 

Although many studies have shown a shift toward a dyslipidemic profile, but results are not consistent, and investigators generally have not adjusted for possible confounding factors such as age, BMI, and gestational age (13). The present study investigates the relationship of plasma lipids (cholesterol, triglycerids), and lipoproteins (LDL-C and HDL-C) levels in preeclamptic and normal overweight pregnant women and compare the results between the two groups.

## Materials and methods

This study was a case-control study for comparison of lipid profile in overweight preeclamptic and overweight normotensive pregnant women. The patients participating in this study were recruited from among those managed at the Alzahra Hospital in Guilan University of Medical Science from March 2008 to October 2009.

Pregnant women were between 16-45 years old with singleton pregnancy and 30-42 weeks of gestational age on the basis of ultrasonographic findings below the 20 weeks of gestation and last menstrual period, and had prepregnancy BMI ≥25. The study was approved by the ethics committee of Guilan University of Medical Science and informed consent was obtained from each subject: forty-two women with singleton pregnancies, with prepregnancy BMI> 25 and with the diagnosis of preeclampsia constituted the study group.

Maternal height was measured without shoes at the time of arrival to hospital and prepregnancy weight was recorded on the based of maternal self report in first trimester of pregnancy and BMI was accounted based on those criteria’s [BMI; weight (kg)/height (m)^2^]. Preeclampsia was defined as blood pressure >140/90 mmHg after 30 min of rest on two separate occasions at least 6hr apart with proteinuria ≥1^+^ (100 mg/dl) on a voided specimen and ≥ 300 mg/24hr in the presence of no urinary infections.

Preeclamptic women were further subdivided as mild and severe according to the criteria of American College of Obstetricians and Gynecologists (14). Severe preeclampsia was defined by criteria of BP≥160/110 mmHg after 20 weeks gestation, proteinuria 2.0g/24 hours or >2^+^ dipstick, serum creatinine >1.2 mg/dl unless known to be previously elevated, platelets <100,000/ mm^3^, microangiopathic hemolysis (increased LDH), elevated ALT or AST, persistent headache or other cerebral or visual disturbance, and persistent epigastric pain (2). 

Forty-one healthy normotensive pregnant women with prepregnancy BMI≥25 made up the control group. The subjects were between 30 and 42 gestational weeks and were between 16-45 years old at the time of sampling. 

Women with underlying chronic illness (renal, hepatic, endocrine such as hypothyroidism or diabetes mellitus and cardiovascular, with acute or chronic infection), receiving any drugs interfering with lipid metabolism (Erythromicin, Nitrates, Statines, Steroides, B blockers, Phenytoin, Sulfonamides, Thiziedes), the subjects were suspicious for familial hyperlipidemia: (TG> 500 or cautenous xantoma or tendinus or Altherosclerosis at age <20 yrs), whose fetuses had any congenital anomalies based on sonographic findings or who had multiple gestation, were unhealthy at discharge from the hospital were excluded from the study. 

All of the subjects were free of medical complications before the onset of preeclampsia. The normotensive overweight pregnant women were matched for age groups to <20 yrs, 21-30 yrs, 31-40 yrs and >40 yrs and for gestational age to 30-36 weeks and >36 weeks with preeclamptic women. Peripheral venous blood samples were collected in to hospital at the time of first diagnosis of preeclampsia before any drug administration. 

None of the patients were in labor phase at the time of blood sampling. Blood was collected early in the morning after a 14-h fast. Blood pressure was also recorded just before sampling. Blood was obtained by venipuncture in test tubes without anticoagulant and serum was taken from that. Those women with severe preeclampsia that were candidate for urgent termination of pregnancy excluded from the study. Because, we didn’t have enough time for 14hr fasting before blood sampling. 

Maternal fasting blood samples collected in 10-ml tubes and were frozen at -80^O^C until analysis. Enzymatic colorimetric test was used to define serum triglyceride, total cholesterol, and Low-density Lipoprotein (LDL) cholesterol (GPO-PAP, CHOD-PAP, and LDL-C method respectively; Parsazmun Co kits, Iran/ Auto analyzer RA100, USA) by Analyzer BT2000. High-density lipoprotein (HDL) cholesterol was determined by detergent-based isolation and enzyme-linked colorimetric detection (CHOD-PAP; Parsazmun Co kits, Iran/ Auto analyzer RA100, USA). 

In this case-control study, we selected 42 overweight women who experienced preeclampsia and 41 healthy overweight pregnant women as control group. The normality of continues data checked by one-sample Kolmogorov-Smirnov test and compared between groups with independent T-Test and one-way ANOVA and post HOC Tukey HSD test as needed.

**Statistical analysis**

The categorical outcome variables were compared with Chi-square test. The odds ratio of the association between preeclampsia risk and maternal plasma lipid concentrations and corresponding 95% CI were calculated using 2×2 Chi-square test. The statistical significance was set at 0.05 levels. SPSS V.16 was used for statistical analyses. 

## Results

The clinical characteristics of the present study's subjects are given in Table I. The two groups were comparable with respect to mean maternal age, mean gestational age, and body mass index. Table II shows plasma lipid concentrations in the two groups, preeclamptic patients were compared with noromotensive pregnant women. In the subjects with preeclampsia, serum triglyceride and cholesterol levels significantly increased and plasma HDL-cholesterol concentrations decreased compared with the controls, (p<0.05), but plasma LDL cholesterol levels didn’t differ between the two groups. We estimated the risk of mild and severe preeclampsia in relation to increasing serum levels of triglyceride, total cholesterol, LDL-C and HDL-C. 

Maternal plasma lipid and lipoprotein concentrations showed significantly differences depending on severity of preeclampsia (Table III). Women who developed severe preeclampsia had higher concentrations of TG and cholesterol and lower levels of HDL compared to noromotensive group. TG: 375.16 vs. 202.85, p<0.001, cholesterol: 245.64 vs. 214.32, p=0.04, HDL: 40.80 vs. 48.95, p=0.03). There were no differences in LDL level between the three groups (mild, severe preeclampsia and noromotensive patients). 

Table IV lists results from Chi-square analyses in which we estimated the odds ratios of preeclampsia associated with varying concentrations of maternal plasma lipids and lipoproteins. There was a 2.75-fold increase in risk of preeclampsia among women with triglyceride concentrations> 175 mg/dl, as compared with women whose triglyceride concentrations were <100 mg/dl.

**Table I:** Characteristics of study subjects according to preeclampsia status

**p-value**	**Normal group (n=41)**	**Preeclampsia group (n=42)**	**Characteristics**
0.89	28.27 (5.75)	28.45 (6.67)	Maternal age (y)
0.24	36.35 (25.25)	35.69 (2.74)	Gestational age (week)
0.18	28.66 (3.32)	27.83 (2.20)	BMI (Kg/m^2^)

The results are presented as mean values ± SD.

**Table II:** Maternal plasma lipid and lipoprotein concentrations according to preeclampsia status

**p-value**	**Normal group (n=41)**	**Preeclampsia group (n=42)**	**lipid concentrations**
0.001	202.85 (63.27)	340.29 (106.45)	Triglyceride
0.02	48.95 (9.48)	43.12 (12.00)	HDL
0.15	124.07 (40.27)	138.24 (47.34)	LDL
0.02	214.32 (42.12)	238.31 (49.65)	Total cholesterol

The results are presented as mean values ± SD.

**Table III:** Blood lipids in normal pregnant and preeclamptic patients

**Parameters**	**Normal blood pressure**	**Mild preeclampsia**	**Severe preeclampsia**	**Statistical relation with group A**
Triglycerides (mg/dl)	202.85 (63.28)	289.00 (68.00)	375.16 (114.67)	p=0.052 in group B p<0.001 in group C
HDL (mg/dl)	48.95 (9.48)	46.53 (11.41)	40.80 (12.06)	p=0.987 in group B p=0.03 in group C
LDL (mg/dl)	124.07 (40.27)	120.41 (48.62)	150.36 (43.30)	p=0.782 in group B p=0.076 in group C
Total cholesterol (mg/dl)	214.32 (42.12)	227.53 (56.08)	245.64 (44.45)	p=0.905 in group B p=0.042 in group C

All the parameters in this table are expressed in Mean±SD with ANOVA and POST HOC test (Tukey).

**Table IV:** Odd ratio (OR) and 95% confidence interval (CI) of the association between the risk of preeclampsia and maternal plasma lipid concentration

**Lipid concentration (mg/dl)**		**Preeclampsia (n=42)**		**Normotensive (n=41)**		**OR (95% CI)**
		**N**	**%**	**N**	**%**	
Total cholesterol						
	<172	7	17.1	4	9.5	Referent
	172-205	10	24.4	8	19	1.4 (0.3-6.53)
	>205	24	58.5	30	71.4	2.18 (0.57-8.38)
LDL cholesterol						
	<83.3	8	19.5	5	11.9	Referent
	83.3-108	6	14.6	7	16.7	1.86 (0.39-8.89)
	>108	27	65.9	30	71.4	1.77 (0.51-6.09)
HDL cholesterol						
	>50	12	29.3	8	19	Referent
	40-50	21	51.2	16	38.1	3.37 (1-11.45)
	<40	8	9.5	18	42.9	1.14 (0.37-3.45)
Triglycerides						
	<100	1	2.4	0	0	Referent
	100-175	16	39	0	0	-----
	>175	24	58.5	42	100	2.75 (1.99-3.78)

Chi-square- OR and 95% CI.

## Discussion

The results showed that dyslipidemia, particularly hypertriglyceridemia are a strong independent risk factor for preeclampsia in overweight pregnant women, and may be an important mediator of the BMI-preeclampsia association.

These findings are inconsistent with previous reports showing that women who develop preeclampsia have higher fasting triglyceride, cholesterol and LDL-C plasma levels and lower HDL-C concentrations than controls (3, 15).

While maternal obesity is an independent and strong risk factor for preeclampsia, less is known about its mechanisms and the relationship between maternal prepregnancy overweight, dyslipidemia, and the risk of preeclampsia (15), but these conditions might be related through common features related to oxidative stress and altered vascular function. The incidence of obesity is rapidly increasing worldwide, and it does the incidence of preeclampsia will undoubtedly increase (16). 

Therefore, understanding mechanisms underlying the BMI-preeclampsia relation is of great public health importance. Few studies have examined the association between prepregnancy overweight and dysregulation of metabolic pathways during pregnancy (3, 16). In normal pregnancy the circulating concentrations of triglyceride and cholesterol are increased progressively to supply the developing fetus (17). 

In women with preeclampsia the serum lipids show a shift towards a dyslipidemic profile (15). Altered lipid synthesis and abnormal lipid metabolism seems to be important in the pathogenesis of preeclampsia (5), and the role of hypertriglyceridemia and high LDL-C levels in pathogenesis of preeclampsia is confirmed in majority of studies (3, 5, 10, 15). Investigators have suggested that dyslipidemia may contribute to the increased oxidative stress and endothelial dysfunction observed in preeclampsia (18). Dyslipidemia may impair trophoblasts invasion thus contributing to a cascade of pathophysiologic events that lead to the development of preeclampsia. This hypothesis is supported by the fact that triglyceride accumulation in endothelial cells is associated with decreased release of prostacyclin (18).

Documentation of an association between obesity, dyslipidemia and preeclampsia has public health and clinical significance, because the high prevalence of overweight and obesity, suggest the vulnerability of women of reproductive years to hypertensive disorders of pregnancy.

There is likely more than one mechanism underlying the relation of prepregnancy overweight to preeclampsia. One possible mechanism about the association between prepregnancy high BMI is dyslipidemia. Obesity and preeclampsia share many common features. Both of them are associated with oxidative, stress, dyslipidemia, hyperinsulinemia, insulin resistance and impaired endothelial function (19). 

Women with a history of preeclampsia, as compared with their BMI-matched counterparts without such a history, have higher circulating levels of fasting insulin and lipids, years after delivery. Also individuals with the metabolic syndrome, of which obesity is a major feature, also exhibit chronic hypertriglyceridemia, a risk factor for endothelial dysfunction (20). Thus the increased risk of preeclampsia in overweight women may be via the metabolic syndrome (21), and metabolic syndrome and related vascular disorders may also be important in determining the occurrence of preeclampsia (22).

A few studies have evaluated the relationship between plasma lipid concentrations and severity of preeclampsia (23, 11-13). The degree of obesity is related to the severity of preeclampsia, and women with the highest BMI are at increased risk of severe preeclampsia (24). In a study assessing the risk factors for severe preeclampsia, other than a history of preeclampsia, severe obesity (BMI≥ 32.2) was the only other maternal risk factor that was identified for the development of severe preeclampsia (25). 

In one study by Akhavan *et al* the association between hyperlipidemia and severity of preeclampsia was evaluated and patients with severe preeclampsia had significant increase in plasma triglyceride, cholesterol, and LDL–C levels compared with controls (11). These findings are in agreement with our results. We found increase plasma triglyceride, (p<0.001) and cholesterol (p=0.04(, and lower HDL-C (p=0.03( concentrations in severe preeclampsia compared with normotensive pregnant women, but plasma LDL levels were not different between the two groups. In our study severe preeclampsia risk was 2.75 fold of increase among individuals having triglyceride levels above 175 mg/dl comparing those under 100 mg/dl (95% CI 1.99-3.78). 

On the other hand in a prospective study, Baker *et al* found that women with the most severe form of preeclampsia had TG levels similar to normotensive control subjects. They concluded that there may be variants of preeclampsia that can be identified by differences in their lipoprotein profiles and different pathological process between mild and severe preeclampsia (12). Their findings are not comparable with our result, because in their study the serum samples were collected at midgestation before clinical manifestation of preeclampsia and from a non fasting state, which could alter the results.

Our study had some limitations that merit discussion. Collection of blood samples to assess plasma lipids was in third trimester in women presenting with clinical manifestation of preeclampsia in our study and in first trimester in the most studies that would be noticeable in interpretation of our results (3, 11, 12, 15). 

Second, single measurement of lipid profile may lead to some misleading data and serial measurement is recommended in longitudinal studies to determine their change during pregnancy.

Third, as with any case-control study, there is potential for selection bias. Although we attempted to match for as many covariates as feasible, there is still a possibility that we did not account for all of them. Important strengths of this study include the demographics similarities between the groups and similar maternal age and gestational ages of groups at serum collection. Also we excluded all women with chronic medical diseases as they can have impact on lipid levels. Our study was different in that all of subjects were overweight (BMI>25) at the start of pregnancy. 

In this study we noted that dyslipidemia; particularly hypertriglyceridemia was highly correlated with prepregnancy high BMI in preeclamptic women. These findings continue to support a role for dyslipidemia in BMI related preeclampsia and may be of etiologic and pathophysiologic importance in this relatively common complication of pregnancy in overweight and obese women. 

If our results are confirmed by other investigators, these data suggest that interventions designed to reduce triglyceride concentrations at the start of pregnancy may be effective in lowering the risk of preeclampsia among overweight and obese women. Knowledge from such studies may contribute to the development and evaluation of behavioral and medical interventions aimed at reducing medical complications of pregnancy and improving the health of reproductive-aged women. However it is not known whether a program of life style modification, including prepregnancy weight loss, and antilipid drugs can reduce the risk of preeclampsia. Randomized clinical trials might usefully evaluate these questions among obese women of reproductive age. Investigation is crucial to determine if these abnormal lipid concentrations observed in women with preeclampsia are present pre-pregnancy or develop as a result of the disease process of preeclampsia. Further investigation is recommended to determine persistence of this abnormality post-pregnancy and their contribution in long-term cardiovascular diseases.
